# Onchocerciasis then and now: achievements, priorities and challenges

**Published:** 2018-02-08

**Authors:** Adrian Hopkins

**Affiliations:** 1 Consultant. Former Director of the Mectizan Donation Programme. Gravesend, UK.

**Although river blindness is still endemic in many African countries, it is still possible that it will be eliminated by 2025. Doing so will require political stability and an unwavering focus on the goal.**

**Figure:**
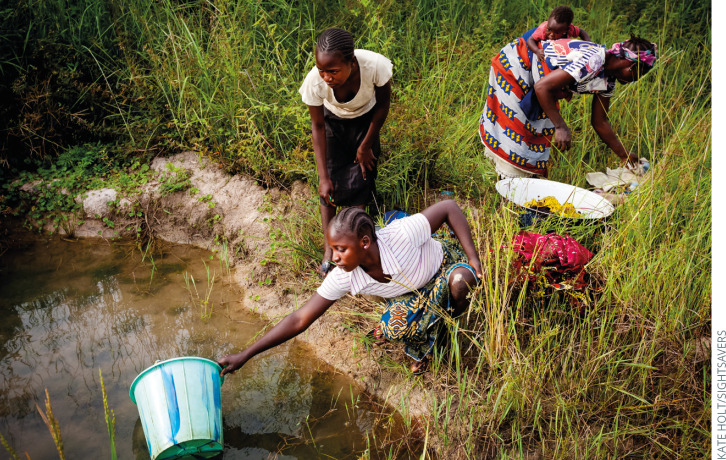
The black fly, responsible for onchocerciasis transmission, breeds along rivers such as these. NIGERIA

Onchocerciasis is an eye and skin disease caused by a worm known as *Onchocerca volvulus.* It is transmitted from one human to another by black flies of the genus *Simulium.* It causes an itchy skin rash, eye disease (often blinding) and nodules under the skin. More than 99% of the people with this infection live in Africa.

In 1987, just before first issue of the *Community Eye Health Journal* was published, the pharmaceutical Company MSD (known as Merck & Co. Inc. in the USA and Canada) made an unprecedented commitment to donate Mectizan^®^ (ivermectin MSD), for as long as was needed, to control onchocerciasis (river blindness).^1^ Mass distribution of Mectizan revolutionised the approach to onchocerciasis control at the time, and has since led to mass drug administration for some of the other neglected tropical diseases (NTDs). It had become possible to imagine that onchocerciasis would one day be eliminated.

**Figure 1:**
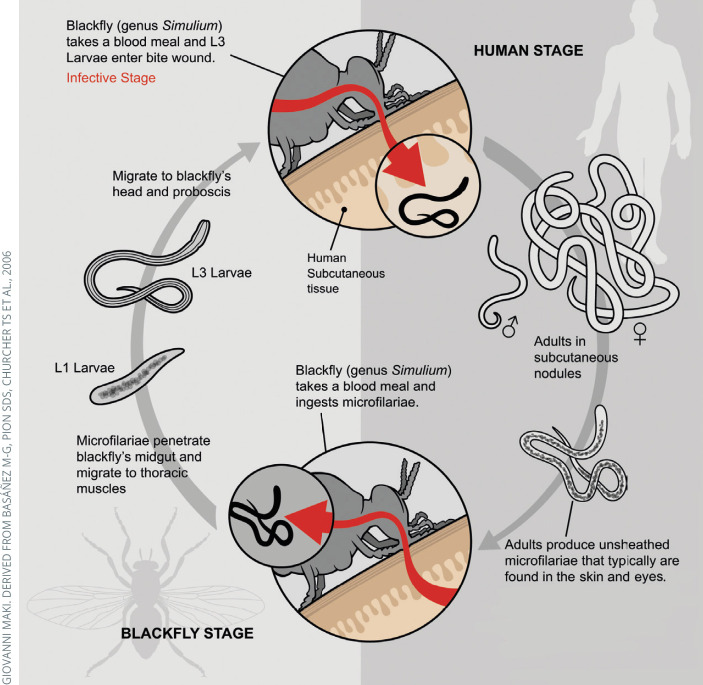
Life cycle of *Onchocerca volvulus*

## The Onchocerciasis Control Programme

The first very successful, but very costly, control efforts had begun more than a decade earlier, in West Africa. These efforts were led by the Onchocerciasis Control Programme (OCP) in West Africa. The only tool available at the time was vector control: limiting or eradicating the insect responsible for spreading onchocerciasis.^2^

The principle was that, by controlling breeding sites of the flies for long enough, transmission could be interrupted while waiting for the adult parasites of *Onchocerca volvulus* to die (in infected individuals). Then, even if the flies returned, there would be no further larvae to transmit. This required the regular spraying of larvicide along thousands of kilometres of rivers in West Africa at very regular intervals during the peak breeding season, often using helicopters to treat inaccessible areas. Although transmission was in fact halted in many areas, nothing could be done for those patients already suffering from the effects of the disease which – as well as visual loss – included skin disease with severe itching.

## Natural history

The adult stage of *Onchocerca volvulus* is a worm. In the bodies of humans, the worms are found intertwined in nodules, most of which are found on bony prominences just under the skin. These, however, cause few problems. However, each female adult worm produces thousands of larvae (microfilaria) which create the major problems of the disease.

The microfilariae migrate around the body, with most going to the skin where they are ingested with the blood meal during a black fly bite (and passed on to others when the same black fly bites them later). Microfilariae also find their way to other parts of the body, including the eye.

When the microfilariae in the tissues are alive, they cause few problems. Those which are not ingested by a black fly within six months or more will die, which causes a localised inflammatory response. In the skin this provokes itching which can be very severe.

The inflammatory response can also occur in any structure of the eye or optic nerve. Inflammatory reactions around an individual microfilaria are usually insignificant, but repeated sites of inflammation around many dying microfilaria eventually create irreversible changes. In the eyes, the most significant lesions causing visual impairment and blindness are sclerosing keratitis, anterior uveitis, chorioretinitis and optic atrophy.

## Treatment

Diethylcarbamazine(DEC), known under various trade names in Africa, has been around for many years. It kills microfilaria but may lead to severe reactions in heavily infected patients. These reactions are called Mazzoti reactions and are due to dying microfilariae. In the eye, the inflammation is often so intense that it actually creates further visual loss and so use of DEC is contraindicated. Ivermectin may also lead to Mazzoti reactions, but these are much less severe and only occur in heavily infected individuals.

Ivermectin was shown to have no effect in the eye. The microfilariae are killed in the skin. Those in the eye either die naturally in the eye, or migrate out of the eye. There is therefore no detriment to vision when using ivermectin, although most changes that are already present are irreversible.

## Ivermectin and onchocerciasis control

With the availability of ivermectin, a new era of safe, effective treatment for patients and disease control began. Ivermectin was first introduced in West Africa, where the Onchocerciasis Control Programme had been active. The donation also presented an opportunity to treat other areas. Eye care nongovernmental development organisations (NGDOs), which had established blindness rehabilitation programmes in onchocerciasis endemic areas, were among the first to start distribution programmes in highly endemic areas. These NGDOs met together with the World Health Organization (WHO) to select priorities and coordinate programmes and develop common approaches to treatment.

Ivermectin kills microfilariae but not the adult worm. It does have a temporary sterilising effect on the adult females, thereby delaying by several months the re-invasion of the skin and other tissues. Early studies showed that an annual dose of ivermectin would control the symptoms of the disease and would prevent the development of further vision loss. Endemic areas were classified as hyperendemic, mesoendemic and hypoendemic. There were very few cases of blindness (or skin disease) in hypoendemic areas and these were not considered priority areas for control programmes. Endemicity was initially measured by skin snip studies where live microfilariae were visible in small pieces of skin.

As experience grew with the use of ivermectin, it was found possible to simplify the community diagnostic process using a nodule survey. If the prevalence of nodules was 20% or more, a community was considered meso- or hyper-endemic and received ivermectin. In these communities, all eligible people were treated thanks to mass drug administration (MDA) projects. Certain groups were excluded: children younger than five, pregnant women, lactating women during the first week post-partum, and those with a chronic disease, particularly diseases of the central nervous system. Although ivermectin was donated and imported to the government stores free of charge, there were still considerable costs and logical challenges. The costs related to training staff for ivermectin distribution and there were logistical challenges to get the medicines to the most remote areas, where most of the patients were found.

## The African Programme for Onchocerciasis Control

The biggest problem was in Africa, which had over 99% of the global burden. It soon became clear that NGDOs working with national ministries of health would be unable to scale up as required. Negotiations with all the partners involved led to the creation of the African Programme for Onchocerciasis Control (APOC) in 1995. The programme was largely financed by donors to a World Bank Trust Fund, with the WHO as the implementing agency, supported by governments and NGDOs.

## Operational research

Research by the WHO's Special Programme for Research and Training in Tropical Diseases (TDR) brought about some major developments for onchocerciasis control. These included mapping and the involvement of communities in managing their own programmes of community-directed treatment with ivermectin (CDTI). The social consequences of severe skin disease were also identified as a problem of serious public health importance.

**Figure:**
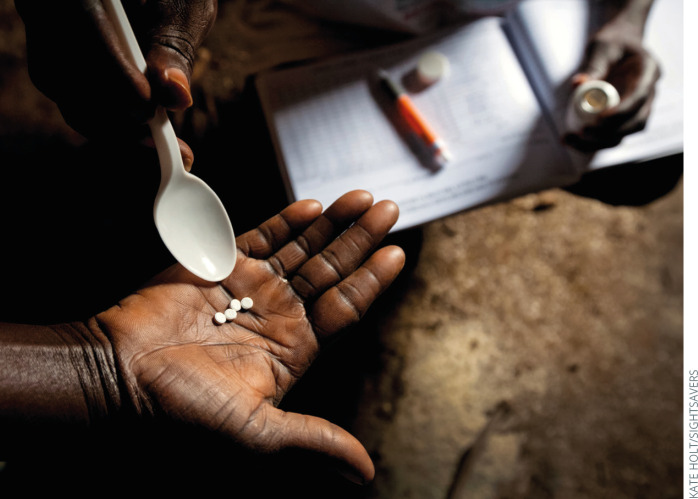
Community distribution of ivermectin for onchocerciasis. NIGERIA

**Figure 2:**
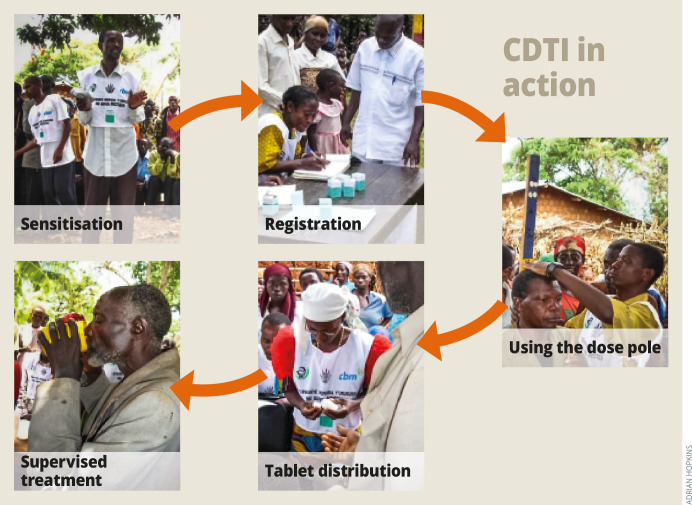
Community-directed treatment with ivermectin

Mapping techniques started to develop rapidly when it was discovered that there was an approximate relationship between the onchocercal nodules under the skin and the prevalence of skin microfilaria. In Africa, mapping techniques evolved from nodule surveys in each village, to a system of rapid epidemiological mapping for onchocerciasis (REMO). After careful selection of a limited number of communities, their populations were assessed for nodules, and regions of meso and hyperendemic onchocerciasis defined. After the populations were made aware of onchocerciasis and its treatment, and chose people to train as distributors of ivermectin, they carried out their own MDA and reported the results. With the resources of APOC and support from NGDOs, governments were able to map onchocerciasis using this REMO technique. Efforts were scaled up over a period of a few years so that most endemic areas were covered.

There were a few problem areas, notably in conflict or post-conflict areas, and in co-endemic areas with another filarial disease, Loa loa. APOC was however a major success as a control programme, as acknowledged in the final evaluation in 2015.^3^ A total of 120 million people were treated. Communities were taking ownership of not only the onchocerciasis programme, but also mass drug administration for other neglected tropical diseases. Onchocerciasis had been largely eliminated as a public health problem. There are probably only a few new cases of blindness from onchocerciasis in Africa every year, mainly in areas of political instability where programmes have not been functioning well.

## Onchocerciasis Elimination Programme for the Americas

In Latin America, the areas affected by onchocerciasis were small and well circumscribed, with an estimated half a million people infected. Here, the strategy was to treat everyone at risk and not to take into account the levels of endemicity.

The Onchocerciasis Elimination Programme for the Americas (OEPA) set out with the objective of eliminating the transmission of onchocerciasis. In 1995, some areas were put on twice yearly treatment with ivermectin. From 2000, there was a big effort to scale up treatment and achieve excellent coverage. The result has been that onchocerciasis has been eliminated in Colombia, Ecuador, Mexico and Guatemala. Only Venezuela and Brazil still have some cases, particularly deep in the Amazon forest, on their common border.^4^

## Elimination of transmission in Africa: a paradigm shift

In Africa, where the epidemiology is completely different, with large foci often not well defined and treatment focused primarily on control of disease as a public health problem, the main question was if, and when, treatment with ivermectin could be stopped. Studies in Senegal and Mali (completed in 2009) showed that transmission of the disease had been interrupted by multiple rounds of annual ivermectin treatment. Elimination of transmission was now a possibility and on the agenda for Africa.

In 2009, the first consultation between partners took place to review the possibilities.^5^ At the annual meeting of APOC (the Joint Action Forum) it was agreed to change the focus of the control programmes to the elimination of transmission, where possible. This shift needed to be reflected in country programmes. WHO has now produced updated guidelines on criteria for elimination^6^, but countries still need help to reach this goal; for example, there must be mapping for elimination that includes all potential hypoendemic regions.

Decisions must be made about alternative treatments for many of the affected areas. For example, whether treatment should be increased to twice or even four times a year in some areas, and whether short-term vector control should be added in some areas to break the transmission cycle. These decisions must be made at the national level, and many countries are now setting up ‘elimination expert advisory committees.’ For example, in Uganda, which has had a committee in place for 10 years, some areas are now clear of the disease and treatment has been stopped. However, due to conflict in the north of the country, the programme has only been carried out at full scale over the last two to three years. Cross-border areas continue to cause difficulties in Uganda, due to delayed programmes in the DRC and South Sudan.

## Future opportunities

Whereas much has been achieved using existing control strategies, more is needed to enhance diagnosis and treatment. For example:

- Developing on an easy-to-use macrofilaricide, which would kill the adult parasite, would have a major impact on speeding up the elimination process.
- Skin snips have started to be replaced by serological tests, using the OV-16 antibody test, and this could be implemented in more areas.
- Ongoing research about the best strategies to use in areas where loiasis is co-endemic has had some success, but further research is needed to see how to put the strategies into policy, particularly in Cameroon and the DRC where the problem is greatest.
- Ivermectin is also used for mass drug administration in the lymphatic filariasis (LF) elimination programme in Africa, where the two diseases co-exist. Strategies must be coordinated and a process for monitoring and evaluation needs to be established, especially where one disease may have already been eliminated but treatment needs to continue for the other.

**Figure 3:**
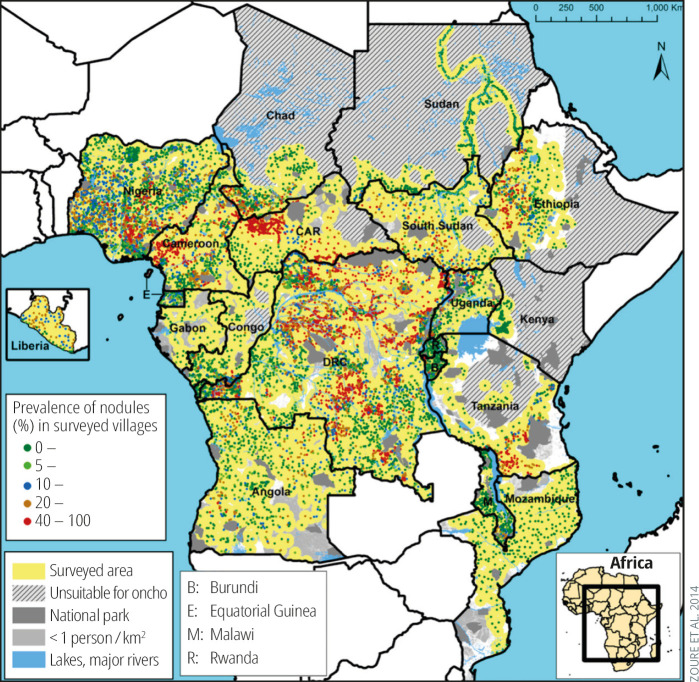
The baseline endemicity of onchocerciasis in APOC countries.

The international community has set a target for elimination of onchocerciasis by 2025. This is ambitious but possible, provided there is commitment and political stability in Africa and Yemen, which is working on its own elimination programme. As endemic countries establish their health targets for the future, onchocerciasis elimination must be a priority.

**Figure 4:**
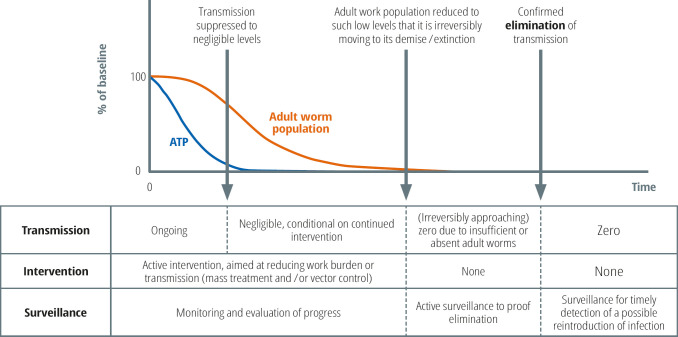
Phases of elimination

## References

[1] The Mectizan Donation Programme. **http://www.mectizan.org/about/history** Accessed 7 September 2017.

[2] Boatin B. The Onchcerciasis Control Programme in West Africa (OCP). Ann Trop Med Parasitai 2008;102(Suppl 1):13–17. 10.1179/136485908X337427 18718148

[3] Fobi G Yameogo L Noma M Aholou Y Koroma JB Zouré HM, et al. (2015) Managing the Fight against Onchocerciasis in Africa: APOC Experience. PLoS Negl Trop Dis 9(5):e0003542. 25974211 10.1371/journal.pntd.0003542 PMC4431813

[4] Gustavsen K Hopkins A Sauerbrey M. Onchocerciasis in the Americas: from arrival to (near) elimination. Parasit Vectors. 2011;4:205. 22024050 10.1186/1756-3305-4-205 PMC3214172

[5] Informal Consultation on Elimination of Onchocerciasis transmission with current tools in Africa – “Shrinking the Map”. **https://tinyurl.com/oncho-eliminate** Accessed 7 September 2017.

[6] Onchocerciasis: Guidelines for stopping mass drugadministration and verifying elimination of human onchocerciasis. **https://tinyurl.com/Oncho-Guide.** Accessed 4 December 2017.

